# Factors associated with 10 years of continuous viral load suppression on HAART

**DOI:** 10.1186/s12879-016-1677-x

**Published:** 2016-07-22

**Authors:** Kathryn J. Bello, Octavio Mesner, Thomas A. O’Bryan, Seung Hyun Won, Tahaniyat Lalani, Anuradha Ganesan, Brian K. Agan, Jason F. Okulicz

**Affiliations:** Internal Medicine Service, San Antonio Military Medical Center, 3551 Roger Brooke Drive, Fort Sam Houston, 78234 TX USA; Infectious Disease Clinical Research Program, Uniformed Services University of the Health Sciences, 4301 Jones Bridge Road, Bethesda, 20814 MD USA; Infectious Disease Service, San Antonio Military Medical Center, 3551 Roger Brooke Drive, Fort Sam Houston, 78234 TX USA; Naval Medical Center, 620 John Paul Jones Circle, Portsmouth, 23708 VA USA; Infectious Disease Service, Walter Reed National Military Medical Center, 8901 Rockville Pike, Bethesda, 20889 MD USA; Henry M. Jackson Foundation for the Advancement of Military Medicine, 6720A Rockledge Drive #100, Bethesda, 20817 MD USA

**Keywords:** HIV, AIDS, Viral load, Suppression, HAART, CD4 cell count

## Abstract

**Background:**

The principal goal of HAART is sustained viral load (VL) suppression resulting in immune reconstitution and improved HIV outcomes. We studied the factors associated with 10 years of continuous VL suppression on HAART in the US Military HIV Natural History Study.

**Methods:**

Participants with continuous VL suppression (CS, *n* = 149) were compared to those who did not have continuous viral load suppression (NCS, *n* = 127) for ≥10 years on HAART. Factors associated with >10 years of VL suppression were evaluated by multivariate logistic regression. Additionally, association between CS and CD4 reconstitution was analyzed with a mixed effects model.

**Results:**

Compared to NCS participants, a lower proportion of CS participants started HAART in the early HAART era (66 vs 90 %, for years 1996–1999; *p* < 0.001) and had less antiretroviral use prior to HAART (37 vs 83 %; *p* < 0.001). At initial HAART, the median CD4 cell count was higher and VL was lower for CS compared to NCS participants (375 cells/uL [256, 499] vs 261 cells/uL [146, 400]; *p* < 0.001 and 4.4 log_10_ copies/mL [3.5, 4.9] vs 4.5 log_10_ copies/mL [3.8, 5.0]; *p* = 0.048, respectively). New AIDS events were lower during HAART (5 vs 13 %; *p* = 0.032) and post-HAART CD4 trajectories were greater for the CS compared to NCS group. Factors negatively associated with ≥10 years of VL suppression included log_10_ VL at first HAART (OR 0.61, 95 % CI 0.4, 0.92; *p* = 0.020) and antiretroviral use prior to HAART (OR 0.16, 95 % CI 0.06, 0.38; *p* < .001).

**Conclusions:**

Sustained VL suppression is a key to long-term health in HIV-infected patients, as demonstrated by the lower proportion of AIDS events observed 10 years after HAART initiation. The current use of more potent and well-tolerated regimens may mitigate the negative factors of pre-HAART VL and prior ARV use encountered by treatment initiated in the early HAART era.

## Background

The primary goal of highly active antiretroviral therapy (HAART) in HIV-infected individuals is sustained viral load (VL) suppression which, in turn, leads to several benefits. Immune recovery is evidenced by CD4 cell gains, a reduction in activated CD8 cells, decreased immune activation, and enhanced responsiveness to vaccines [1–3]. Effective HAART can also mitigate opportunistic infections and the development of acquired immunodeficiency syndrome (AIDS) events [4]. Successful HIV treatment is also associated with a reduction in HIV-associated non-AIDS diseases, including cancer and cardiovascular, renal, and liver disease [5, 6]. Recent studies have demonstrated life expectancy approaching that of HIV-uninfected persons for those with low rates of virologic failure and relatively preserved CD4 counts [7]. In addition, sustained VL suppression enhances the durability of the treatment regimen and prevents the development of antiretroviral resistance [8].

Unfortunately, not all patients are able to maintain VL suppression on HAART. In the US, approximately 40 % of HIV-infected persons have a suppressed VL [9]. Recent cohort studies report VL suppression rates ranging between 65 and 80 % on a variety of HAART regimens, whereas newer HAART regimens have suppression rates approaching 90 % in clinical trials [10–13]. There are many patient-related and treatment-related factors associated with virologic failure early in the course of HAART. Unlike many other chronic conditions, adherence is of major importance as the probability of VL suppression is diminished in patients with <95 % adherence [14]. In addition, suboptimal adherence can lead to increased drug resistance and reduced quality of life and survival [15, 16].

Sustained VL suppression is essential to achieve the best possible treatment outcomes. Because a significant proportion of patients do not achieve this goal, we examined factors associated with ≥10 years of VL suppression in the US Military HIV Natural History Study (NHS). We hypothesize that both patient and treatment-related factors will be associated with long-term VL suppression, and analyses included medication adherence and characteristics of the early HAART era such as prior therapy with single or dual agents. Longitudinal treatment outcomes spanning the entire HAART era were also analyzed to evaluate CD4 trajectories and development of new AIDS events during long-term HAART.

## Methods

### Study participants

The NHS is a large prospective multicenter cohort of HIV-infected individuals. The study population is comprised of active duty military, retired military, and their beneficiaries from the Army, Navy/Marines, and Air Force. Participants are evaluated approximately every 6–12 months at military treatment facilities throughout the United States. Data between study visits are systematically collected, including demographic characteristics, laboratory data, information on medication use, and reports of clinical events with medical record confirmation. The NHS has been enrolling since 1986 and consists of more than 5800 participants. Self-reported adherence (SRA) data has been systematically collected since 2006. All participants provided informed written consent and this study protocol was approved by the Uniformed Services University of the Health Sciences central IRB.

### Inclusion criteria

NHS participants initiating HAART on or after January 1, 1996 with ≥10 years of continuous treatment were included. Participants were required to have ≥1 VL and CD4 count determinations per year during follow-up, with data collected through December 31, 2013. Treatment interruptions <6 months in duration were permitted. HAART was defined as two or more nucleoside reverse transcriptase inhibitors (NRTIs) in combination with at least one protease inhibitor (PI), one nonnucleoside reverse transcriptase inhibitor (NNRTI), or one integrase strand transfer inhibitor (INSTI). Regimens comprised of one NRTI with at least one PI and one NNRTI or an abacavir or tenofovir containing regimen of 3 or more NRTIs in the absence of both PIs and NNRTIs were also considered HAART. Non-HAART antiretroviral (ARV) use was defined as treatment not meeting HAART criteria above and typically consisted of mono- or dual-therapy prior to the availability of HAART. The total number of HAART regimens, defined as changes to any or all ARV components, were also captured during the study period.

VL suppression was defined as achieving a VL ≤400 copies/mL within the first year of HAART. Virologic failure was defined as not achieving suppression or two consecutive VL determinations ≥400 copies/mL after initial suppression. The threshold of 400 copies/mL was chosen in order to compare outcomes during the entire HAART era given the differences in VL assay detection limits over time. Participants were divided into two separate groups for analysis. The continuous VL suppression (CS) group was defined as those with all VL values ≤400 copies/mL for ≥10 years. The non-continuous VL suppression (NCS) group was defined as having 1 or more episodes of virologic failure during the study period. Since more sensitive VL assays with limits of detection <50 copies/mL became clinically available during the later HAART era, we also analyzed the proportion with VL suppression <50 copies/mL in both groups. CD4 count trajectories and development of AIDS events after HAART initiation were also analyzed. SRA data was obtained via voluntary participant questionnaire with adherence defined by the overall percentage of HAART doses taken in the prior 6 months.

### Statistical analysis

Cohort demographics and other characteristics associated with HIV were described separately for participants in the CS and NCS groups. For continuous variables, t-tests were used for normally distributed variables or by Wilcoxon tests when appropriate. For categorical variables, chi-squared tests or Fisher’s exact tests were used. From the clinically relevant baseline characteristics, logistic regression was used to analyze the impact of factors on continuous VL suppression during 10 years of HAART. SRA data was calculated as the average and minimum percent adherence and evaluated by t-tests. Adherence was also evaluated as a categorical variable (report of 100 % adherence yes/no) and analyzed by chi-squared test.

Mixed effects models were used to analyze the factors associated with longitudinal CD4 cell reconstitution. The square root of the CD4 count was used to normalize the data. An interaction term between the years from HAART initiation to VL suppression was used to quantify the difference in the rate of CD4 cell increase between groups. From this data subset, a locally-weighted scatter plot smoothing (LOWESS) was created to visualize trends in CD4 cell reconstitution over 10 years on HAART.

## Results

A total of 276 participants met the inclusion criteria (Table 1). Participants were predominately male with similar proportions of Caucasians and African Americans in each group. The median age at HIV diagnosis was 32 and 30 years in the CS and NCS groups, respectively (*p* = 0.065). A greater proportion of NCS participants (90 %) were treated in the early HAART era (1996–1999) compared with the CS group (66 %; *p* < 0.001). The median CD4 count at first HAART was higher in the CS compared with the NCS group (375 vs 261 cells/uL; *p* < 0.001) and the median VL at first HAART was 4.4 log_10_ copies/mL and 4.5 log_10_ copies/mL, respectively (*p* = 0.048). First HAART regimens differed among the groups (*p* < 0.001), with NCS having a higher proportion of unboosted PI use compared with CS participants (76 % vs 51 %). AIDS outcomes prior to first HAART were similar for CS (8 %) and NCS participants (12 %; *p* = 0.399). During longitudinal follow-up, groups had a median of 1 treatment interruption [0,1], with a higher proportion of any treatment interruption observed in NCS (57 %) compared to CS (17 %) participants (*p* < 0.001). NCS participants also had a greater median number of HAART regimens (7 [4, 9] vs 3 [2, 5]; *p* < .001) and a higher proportion of new AIDS events by 10 years of HAART (13 vs 5 %; *p* = 0.032) compared with CS participants. In a subgroup analysis of participants with VL data using assays with a limit of detection <50 copies/mL during HAART, the proportion with a suppressed VL <50 copies/mL was higher in the CS group (2399 of 2550 VL values, 94 %) compared to the NCS group (1545 of 2221, 70 %; *p* < 0.001).

**Table 1:** Baseline Characteristics of Participants

Characteristic	All	Continuous VL suppression	Non-continuous VL suppression	*P*-value
Number of participants, *n*	276	149	127	–
Gender, Male	252 (91 %)	137 (92 %)	115 (91 %)	0.845
Race	–	–	–	0.234
Caucasian	119 (43 %)	70 (47 %)	49 (39 %)	–
African-American	126 (46 %)	61 (41 %)	65 (51 %)	–
Hispanic/Other	31 (11 %)	18 (12 %)	13 (10 %)	–
Median Year of HIV Diagnosis	1993 (1989,1998)	1996 (1991, 2000)	1991 (1987, 1994)	<0.001
Median Age at HIV Diagnosis, years	31 (26,38)	32 (28,38)	30 (25, 37)	0.065
Median Year of HAART Initiation	1997 (1996,1999)	1998 (1997,2001)	1997 (1996,1998)	<0.001
HAART Era at Initiation	–	–	–	<0.001
1996–1999	212 (77 %)	98 (66 %)	114 (90 %)	–
2000–2003	64 (23 %)	51 (34 %)	13 (10 %)	–
Median Age at First HAART, years	37 (32,42)	37 (32,41)	37 (31,42)	0.807
Median CD4 Count at First HAART, cells/uL	319 (200, 458)	375 (256, 499)	261 (146,400)	<0.001
Median VL at first HAART, log_10_ copies/mL	4.5 (3.6,4.9)	4.4 (3.5, 4.9)	4.5 (3.8, 5)	0.048
AIDS before first HAART	27 (10 %)	12 (8 %)	15 (12 %)	0.399
First HAART Regimen	–	–	–	<0.001
3 NRTI	14 (5 %)	12 (8 %)	2 (2 %)	–
Boosted PI	20 (7 %)	7 (5 %)	13 (10 %)	–
NNRTI	53 (19 %)	43 (29 %)	10 (8 %)	–
PI + NNRTI + NRTI	16 (6 %)	11 (7 %)	5 (4 %)	–
Unboosted PI	173 (63 %)	76 (51 %)	97 (76 %)	–
Non-HAART ARV Use Before HAART	160 (58 %)	55 (37 %)	105 (83 %)	<0.001
History of HBV Infection Before First HAART	125 (45 %)	60 (40 %)	65 (51 %)	0.090
History of HCV Infection Before First HAART	12 (4 %)	5 (3 %)	7 (6 %)	0.562

*Abbreviations*: *HAART* highly active antiretroviral therapy, *VL* viral load, *NRTI* nucleoside reverse transcriptase inhibitor, *PI* protease inhibitor, *NNRTI* non-nucleoside reverse transcriptase inhibitor, *HBV* hepatitis B virus, *HCV* hepatitis C virus, *ARV* antiretroviral

### Self-reported adherence

**Fig. 1:**
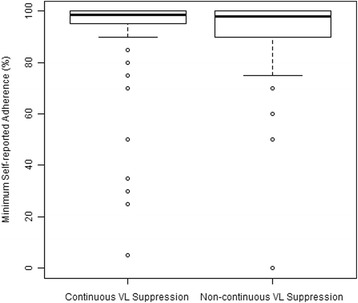
Self-reported Adherence

SRA was high in both groups (Fig. 1), with an average SRA of 99 % in the CS group and 98 % in the NCS group (*p* = 0.341). A similar proportion of participants reported always having 100 % adherence in the CS (26 %) and NCS (24 %; *p* = 0.727) groups.

### Factors associated with VL suppression

Analysis of demographic factors showed no association between age at first HAART and VL suppression for ≥10 years (Odds ratio [OR] 1.0 per 10 years, 95 % confidence interval [CI] 0.68, 1.47; *p* = 0.984, Table 2). The use of non-HAART ARVs prior to first HAART (OR 0.16, 95 % CI 0.06, 0.38; *p* < 0.001) and log_10_ VL at first HAART (OR 0.61, 95 % CI 0.4, 0.92; *p* = 0.020) were both negatively associated with ≥10 years of VL suppression. A negative trend for long-term VL suppression was observed for African Americans compared with Caucasians, but this association was marginal (OR 0.53, 95 % CI 0.25, 1.07; *p* = 0.079). No association was observed for the time from HIV diagnosis to first HAART, CD4 count at first HAART, or type of first HAART regimen. For factors after initiation of first HAART, the total number of HAART regimens used during the study period was negatively associated with long-term VL suppression (OR 0.71, 95 % CI 0.6, 0.81; *p* < 0.001) due to virologic failures, while no association was observed for SRA (OR 0.82, 95 % CI 0.39, 1.73 for those always reporting 100 % adherence; *p* = 0.607).

**Table 2:** Factors Associated with VL Suppression at 10 Years

Characteristic	Odds ratio (95 % CI)	*P*-value
Age at First HAART, per 10 years	1.0 (0.68, 1.47)	0.984
Race (Ref: Caucasian)		
African-American	0.53 (0.25, 1.07)	0.079
Hispanic/Other	0.51 (0.19, 1.42)	0.194
CD4 Count at First HAART, per 50 cells/uL	1.08 (0.99,1.19)	0.112
Log_10_ VL at First HAART	0.61 (0.4, 0.92)	0.02
HAART Regimen (Ref: NRTI)		
Boosted PI	0.8 (0.08, 6.1)	0.841
NNRTI	1.59 (0.18, 9.31)	0.630
PI + NNRTI + NRTI	1.47 (0.15, 11.04)	0.716
Unboosted PI	0.67 (0.08, 3.31)	0.651
Non-HAART ARV Use Prior to HAART	0.16 (0.06, 0.38)	<0.001
Time from HIV Diagnosis to First HAART, months	1.00 (0.99, 1.01)	0.664
Total number of HAART Regimens	0.71 (0.6, 0.81)	<0.001
Self-Reported Adherence Always 100 %	0.82 (0.39, 1.73)	0.607

### CD4 cell reconstitution on HAART

**Fig. 2:**
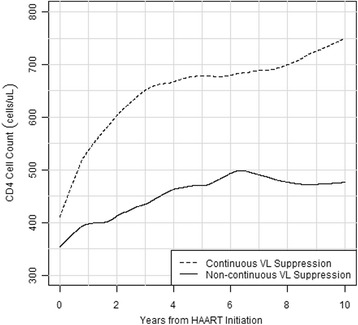
Lowess Curves for CD4 Count Trajectories After HAART Initiation

Longitudinal CD4 cell trends during the first 10 years of HAART showed that the CS group had a greater upward trajectory of CD4 cell gains compared with the NCS group (Fig. 2). Analysis of factors associated with the change in CD4 count from first HAART initiation to 10 years after beginning HAART showed a negative adjusted difference in square root of CD4 (√CD4) for ARV use prior to first HAART (−1.73, 95 % CI−3.44,−0.02; *p* = 0.047), longer time from HIV diagnosis to first HAART (−0.02, 95 % CI−0.03, 0; *p* = 0.025), and higher log_10_ VL at first HAART (−1.7, 95 % CI−2.36,−1.03; *p* < 0.001; Table 3). Continuous VL suppression was associated with greater adjusted difference in √CD4 at baseline (2.3, 95 % CI 0.8, 3.81; *p* = 0.003) and marginal rate of increase in √CD4 during follow-up (0.13 95 % CI 0, 0.26; *p* = 0.049), whereas no association was observed for demographic factors and SRA.

**Table 3:** Factors Associated with Differences in CD4 Count from First HAART to 10 Years

Factor	Adjusted difference in √CD4 from first HAART to 10 years (√cells/uL)	*P*-value
Age at First HAART, per 10 years	−0.38 (−1.06, 0.3)	0.273
Race (Ref: Caucasian)	Reference group: Caucasian	–
African-American	0.19 (−1.06, 1.45)	0.765
Hispanic/Other	0.82 (−1.21, 2.85)	0.429
Log_10_ VL at First HAART	−1.7 (−2.36, −1.03)	<0.001
HAART Regimen (Ref: NRTI)		
Boosted PI	0.23 (−3.25, 3.72)	0.895
NNRTI	0.71 (−2.2, 3.62)	0.631
PI + NNRTI + NRTI	0.27 (−3.39, 3.94)	0.883
Unboosted PI	1.49 (−1.28, 4.26)	0.290
Non-HAART ARV Use Prior to HAART	−1.73 (−3.44, −0.02)	0.047
Time from HIV Diagnosis to First HAART, months	−0.02 (−0.03, 0)	0.025
100 % Med Adherence	0.53 (−0.84, 1.91)	0.445
Continuous VL Suppression	2.3 (0.8, 3.81)	0.003
Rate of Increase for Continuous VL Suppression	0.13 (0,0.26)	0.049

## Discussion

Over the past 20 years, the availability of HAART has effectively transformed HIV from a uniformly fatal condition to that of a chronic disease. Contemporary treatment is associated with improved life expectancy, with one study finding no elevated mortality risk in patients with CD4 counts above 500 cells/uL who maintained VL suppression [17]. Since long-term VL suppression is required for optimal HIV disease outcomes, we aimed to determine the factors associated with sustained virologic control on HAART in a large military cohort.

The inability of many patients to maintain VL suppression can be explained in part by the era in which participants began HAART. A previous study in our cohort reported VL suppression during the early HAART era (1996–2000) compared to the late HAART era (2000–2007), with 58 and 85 % VL suppression at 5 years for those treated in the early versus late HAART era, respectively [10]. Similarly, the Antiretroviral Therapy (ART) Cohort Collaboration analyzed treatment naïve participants who began HAART from 1995 to 2003 and observed an increase in VL suppression in later years, with 58 and 83 % for those initiating HAART between 1995–1996 and 2002–2003, respectively [18]. Our current study with a longer duration of follow-up after HAART initiation (≥10 years) affirms that treatment during the early HAART era is a risk for non-continuous VL suppression.

The use of ARVs prior to HAART, typically mono- or dual-therapy, is an important characteristic of the early HAART era. We observed that ARV use prior to HAART negatively impacted both the ability to achieve long-term VL suppression and the magnitude of CD4 cell gains during HAART. Although non-HAART ARV use was likely a major reason for suboptimal treatment outcomes, other features of the early HAART era other factors likely contributed to the lower rate of treatment success during that period. For example, the early HAART era is also characterized by less potent, unboosted PI regimens, which were more commonly used for first HAART in the NCS group, and other HAART regimens associated with greater adverse effects and reduced tolerability compared to more contemporary regimens [19, 20]. In contrast to the beginnings of HAART, continuous VL suppression is more attainable with newer HAART regimens. For example, recent studies of integrase inhibitor-based regimens have demonstrated VL suppression in approximately 87 % of treatment naïve patients [21, 22]. However, longer follow-up time is necessary to determine the long-term durability of newer regimens.

Advanced HIV disease, as evidenced by low CD4 cell count and AIDS events, is associated with lower rates of VL suppression on HAART [19, 23, 24]. In our current study, we observed that new AIDS events occurring during HAART were more common in those with non-continuous VL suppression despite long-term treatment. We previously found that high cumulative VL on HAART was associated with a greater than 2-fold increase in AIDS events and impacted overall CD4 gains [11]. In our current study, the trajectory of CD4 cells gains over a 10-year period was much higher in those with continuous VL suppression. This is consistent with other studies showing maximal CD4 reconstitution occurs with sustained VL suppression [25, 26]. Thus, the long-term maintenance of VL suppression is of key importance for achieving optimal immune recovery and for the reduction of AIDS events.

Suboptimal treatment outcomes involve both clinical and non-clinical factors. For example, access and engagement in care have been identified as key areas along the HIV care continuum [9]. Some of these barriers are minimized or removed in the NHS cohort as military members and beneficiaries have free access to care and prescription medications. These benefits were demonstrated in a previous NHS study demonstrating a high proportion of VL suppression (81 %) at 1 year [10]. Recent studies in other healthcare settings report slightly lower VL suppression ranging from 70 to 78 %, which may be contributed by access to care or patient characteristics such as injection drug use or socioeconomic factors not typically present in the military population [27–29].

Discordant HIV treatment outcomes have been observed in different racial/ethnic groups. A study of non-white participants found a VL suppression rate of 31 % compared to 54 % in white participants at 7–14 months in an urban setting [23]. Non-white ethnicity was also linked to higher number of missed appointments, injection drug use, and lower CD4 count at HAART initiation [23]. A previous study in our cohort showed that African Americans had lower odds of VL suppression at 6 and 12 months post-compared to participants of European ancestry, although time to virologic failure was no different once suppression was achieved [30]. Similarly, a negative trend for ≥10 years of VL suppression was also observed in African Americans in our current study, however only a small subset of the NHS cohort was included and additional studies are needed to evaluate potential differences according to race/ethnicity.

Adherence is a cornerstone of successful HIV treatment. We found no difference in those reporting 100 % SRA in CS and NCS participants and the overall adherence was 98 % or greater in both groups. This high degree of SRA may be somewhat explained by free access to care and medications, or possibly by other characteristics of our population such as level of education, socioeconomic status, or very low rate of injection drug use [31]. Other explanations may include potential disadvantages of SRA compared with other methods to assess adherence. For example, SRA is subject to recall and social desirability bias and patients tend to report a 10–20 % over estimation on adherence when directly compared to data obtained from electronic drug monitoring [32]. Methods such as medication event monitoring system (MEMS) track caps can be more accurate as they report exact date and times of all bottles opening. One study found that high MEMS adherence rates, but not high SRA, was associated with VL suppression [33]. However, MEMS has potential drawbacks of cost and tracking does not always directly correlate with pill ingestion [34].

The strengths of this study include long-term follow-up spanning the entire HAART era in a diverse cohort of patients with free access to care and medications. Limitations included the inability to assess adherence in the early HAART era since SRA data was not systematically captured until 2006. The high level of SRA in our study likely limited the ability to detect an association between adherence and VL suppression, since >95 % adherence is associated with optimal and sustained VL suppression [14]. However, equally high SRA in both groups allowed for the evaluation of other factors associated with ≥10 years of VL suppression in our cohort. Other potential study limitations include the retrospective study design and non-randomized treatment decisions. Differing limits of detection of VL assays used during the 18-year study period, particularly in the early HAART era, may have limited the interpretation of factors associated with continuous viral load suppression. Our results may not be generalizable to women, given the high proportion of males in our cohort, or to populations with other characteristics such as injection drug use.

## Conclusion

Sustained VL suppression is essential to reduce long-term morbidity in HIV-infected patients, as demonstrated by the lower proportion of AIDS events and improved CD4 cell reconstitution after ≥10 years of continuous VL suppression. We identified factors negatively associated with ≥10 years of VL suppression, including high VL at first HAART, prior ARV use, HIV diagnosis in the pre-HAART era, and number of HAART regimens used. It is likely that treatment in the current era, characterized by regimens that are more potent, more convenient, and less toxic, may promote higher success rates and more durable VL suppression in the future.

## Abbreviations

AIDS, acquired immune deficiency syndrome; ARV, antiretroviral therapy; CS, continuous viral load suppression; HAART, highly active antiretroviral therapy; HIV, human immunodeficiency virus; INSTI, integrase strand transfer inhibitor; LOWESS, locally-weighted scatter plot smoothing; NCS, non-continuous viral load suppression; NHS, natural history study; NNRTI, non-nucleoside reverse transcriptase inhibitor; NRTI, nucleoside reverse transcriptase inhibitors; PI, protease inhibitor; SRA, self-reported adherence; VL, viral load
